# Predictive Value of Carotid Distensibility Coefficient for Cardiovascular Diseases and All-Cause Mortality: A Meta-Analysis

**DOI:** 10.1371/journal.pone.0152799

**Published:** 2016-04-05

**Authors:** Chuang Yuan, Jing Wang, Michael Ying

**Affiliations:** 1 Medical Research Center, Changsha Central Hospital, Changsha, Hunan, China; 2 Department of Health Technology and Informatics, The Hong Kong Polytechnic University, Hung Hom, Kowloon, Hong Kong SAR, China; 3 Department of Neurosurgery/Neuro-oncology, Sun Yat-sen University Cancer Center, State Key Laboratory of Oncology in South China, Collaborative Innovation Center for Cancer Medicine, Guangzhou, China; University of Washington, UNITED STATES

## Abstract

**Aims:**

The aim of the present study is to determine the pooled predictive value of carotid distensibility coefficient (DC) for cardiovascular (CV) diseases and all-cause mortality.

**Background:**

Arterial stiffness is associated with future CV events. Aortic pulse wave velocity is a commonly used predictor for CV diseases and all-cause mortality; however, its assessment requires specific devices and is not always applicable in all patients. In addition to the aortic artery, the carotid artery is also susceptible to atherosclerosis, and is highly accessible because of the surficial property. Thus, carotid DC, which indicates the intrinsic local stiffness of the carotid artery and may be determined using ultrasound and magnetic resonance imaging, is of interest for the prediction. However, the role of carotid DC in the prediction of CV diseases and all-cause mortality has not been thoroughly characterized, and the pooled predictive value of carotid DC remains unclear.

**Methods:**

A meta-analysis, which included 11 longitudinal studies with 20361 subjects, was performed.

**Results:**

Carotid DC significantly predicted future total CV events, CV mortality and all-cause mortality. The pooled risk ratios (RRs) of CV events, CV mortality and all-cause mortality were 1.19 (1.06–1.35, 95%CI, 9 studies with 18993 subjects), 1.09 (1.01–1.18, 95%CI, 2 studies with 2550 subjects) and 1.65 (1.15–2.37, 95%CI, 6 studies with 3619 subjects), respectively, for the subjects who had the lowest quartile of DC compared with their counterparts who had higher quartiles. For CV events, CV mortality and all-cause mortality, a decrease in DC of 1 SD increased the risk by 13%, 6% and 41% respectively, whereas a decrease in DC of 1 unit increased the risk by 3%, 1% and 6% respectively.

**Conclusions:**

Carotid DC is a significant predictor of future CV diseases and all-cause mortality, which may facilitate the identification of high-risk patients for the early diagnosis and prompt treatment of CV diseases.

## Introduction

Increased arterial stiffness is associated with the development of atherosclerosis; therefore, it is a surrogate marker for cardiovascular (CV) diseases [1]. Aortic pulse wave velocity (PWV) is recognized as the ‘gold standard’ of arterial stiffness, because it is the most commonly used stiffness parameter for predicting CV diseases in a substantial number of studies [2]. It is commonly measured at the carotid and the femoral arteries using pressure sensors, and it indirectly reflects the regional arterial stiffness [2, 3]. However, pressure sensors are specific devices and are not always available in all clinical centers. Moreover, the pressure waveform or transit distance may not be accurately recorded in patients with metabolic syndrome, obesity, diabetes mellitus, peripheral artery disease, and aortic, iliac or proximal femoral stenosis [2, 3]; thus, it may be difficult to obtain an accurate measurement of aortic PWV in these patients (PWV = distance/transit time, m/s).

In addition to the aortic artery, the carotid artery is also susceptible to atherosclerosis. Local stiffness of the carotid artery is of particular interest in the prediction of future CV events. In contrast to the regional arterial stiffness, which is indirectly reflected by measuring the pulse wave velocity over the arterial segment, carotid stiffness can be assessed, 1) directly by measuring the pulsatile motions of the carotid artery wall; and 2) via the use of widely available and non-invasive imaging modalities, such as ultrasound or magnetic resonance imaging (MRI) [2, 3]. Thus, the assessment of carotid stiffness may not have the limitations of aortic PWV determination in the prediction of CV diseases.

Various stiffness parameters, such as distensibility coefficient (DC), compliance coefficient (CC), index β, Peterson elastic modulus and incremental modulus of elasticity (Einc) or Young’s elastic modulus (YEM), may be used to represent carotid stiffness. Of these parameters, DC is the relative change in the cross-sectional area/diameter during the cardiac cycle for a stroke change in blood pressure, and it is inversely correlated with arterial stiffness. It reflects the intrinsic stiffness of the artery and can be transferred to local PWV using the following formula: PWV = (ρ*DC)^-1/2^, in which ρ is the blood density and is approximately equal to 1 g/cm^3^ [3, 4]. Thus, the use of DC to represent carotid stiffness may have an advantage for parallel comparisons of local and regional arterial stiffness in the prediction of future CV diseases.

A limited number of studies have shown that carotid DC significantly predicts future CV diseases or all-cause mortality [5–10]; however, several studies have failed to demonstrate consistent findings [11–15]. The predictive value of carotid DC remains debated, and the pooled risk ratios (RRs) of CV diseases and all-cause mortality for carotid DC remain unclear. Thus, we conducted the present study to investigate the overall quantitative estimate of the predictive role of carotid DC.

## Materials and Methods

### Literature research

Search terms, including ((carotid)) AND ((stiffness) OR (distensibility) OR (elasticity)) AND ((longitudinal) OR (prospective) OR (follow-up)) AND ((stroke) OR (coronary heart disease) OR (cerebrovascular disease) OR (cardiovascular disease) OR (death) OR (mortality)), were used to identify studies in the PubMed, Embase and Cochrane databases until November 2015. A manual search of the reference lists in all identified relevant publications and relevant review articles was also performed.

### Selection Criteria

Two reviewers, Y. C. and W. J., independently performed the literature search, study selection and data extraction. Meetings were conducted to address disagreements in these processes until a consensus was achieved. In the initial screening, the abstracts and titles were reviewed in Endnote, and the articles were considered for inclusion in the meta-analysis if they reported the following: 1) the incidence of CV diseases or all-cause mortality, including fatal or non-fatal stroke (ischemic or hemorrhagic), CHD (myocardial infarction, coronary artery bypass grafting, coronary angioplasty or angina pectoris), heart failure and peripheral arterial disease, and sudden death (S1 Table), 2) the assessment of carotid arterial stiffness (DC, CC, index β, Peterson elastic modulus or Einc/YEM), and 3) data from an original human study. The full-texts of the relevant articles identified in the initial screening were reviewed for the second screening. The exclusion criteria for eligibility were: 1) not a longitudinal study, 2) only an abstract, or 3) other stiffness parameters as the determinant rather than carotid DC. For cases of multiple publications [6, 8], the articles with the most up-to-date data were included in the meta-analysis if the risk estimate for carotid DC was available.

### Data extraction

If available, risk estimates with adjustments for covariates were used in our analysis. In the 11 studies included in the present meta-analysis, Blacher et al. reported the risk estimates using dichotomous frequency and odds ratio (OR) [5], whereas Barenbrock et al. demonstrated the risk estimates using OR and Kaplan-Meier survival curve [6]. The ORs were transferred to RRs using the following formula: RR = OR/((1-P0)+(OR×P0)) [16], in which P0 represents the proportion of events in the reference group and was calculated using the dichotomous frequency or data extracted from the survival curve (see S2 Table). The other 9 studies reported HRs, which were directly treated as RRs [17].

In the present study, we investigated the pooled RRs of clinical events for the lowest quartile versus the higher quartiles, a 1 standard deviation (SD) decrease or a 1 unit decrease of carotid DC. Most of the studies included in the meta-analysis reported the risk estimates for change in 1 SD of DC [6–12, 14], whereas other studies used various categories of DC (tertile [13] or quartile [5, 15]). The RRs for each category of DC were converted to each other using a previously published method [18]. For example, two studies reported RRs for the lowest quartile of DC [5, 15]. We assumed that DC values were normally distributed, and used the reported mean and SD to estimate the 12.5th and 62.5th percentiles of DC (which corresponded to the midpoints of the lowest and the 3 higher quartiles, respectively). The RRs after conversion were equal to Exp(natural log RR/(difference of DC between the two percentiles/SD of DC)) and Exp(natural log RR /(difference of DC between the two percentiles)) for the change in DC per SD and per unit, respectively. In addition, for the studies that indicated risk estimates for the increase of DC [7, 11, 12, 14], 1/RR was used in the meta-analysis (See S2 Table).

### Statistical analysis

The quality of each included study was assessed using the Newcastle-Ottawa Quality Assessment Scale (NOS) [19]. The pooled RRs of CV events, CV mortality and all-cause mortality for lower DC were individually investigated in the present study (lowest quartile vs higher quartiles, 1 SD decrease and 1 unit decrease, respectively). The I^2^ statistic was used to determine the proportion of the inconsistencies across studies not explained by chance [20]. Cochran’s Q test was performed to measure heterogeneity among the studies [21]. When the heterogeneity was significant (P<0.05), a random-effects model was applied to calculate the pooled RRs [22, 23]. To determine whether a single study, the duration of follow-up, and the quality of the studies and risk estimates affected the pooled RRs, the sensitivity analyses were conducted by removing one study or unfavorable studies (with a follow-up duration <5 years, NOS score <7, or RR derived from OR), and subsequently calculating the combined RRs of the remaining studies. The publication bias was assessed using funnel plot precision (plots of the effect estimates against the sample size) [24], the classic fail-safe N method (number of missing studies that would produce insignificant results) [25], and the trim-and-fill method (the impute of missing studies and recalculation of the pooled risk estimates) [26]. All the data analyses were performed using Comprehensive Meta Analysis (Version 2, Biostat, Englewood, New Jersey).

## Results

### Summary

**Fig 1:**
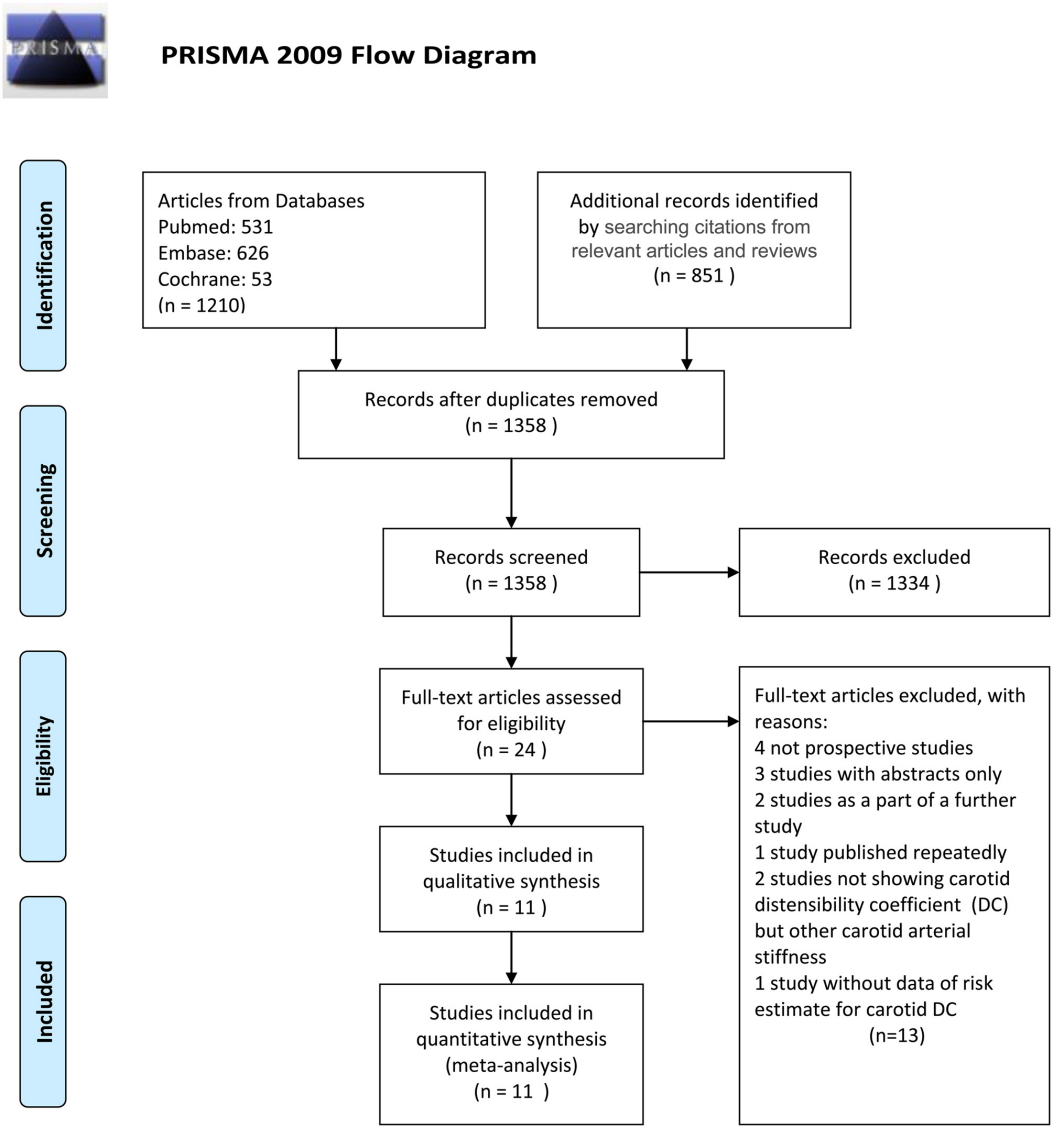
Flow chart for study selection in the meta-analysis. *From*: Moher D, Liberati A, Tetzlaff J, Altman DG, The PRISMA Group (2009). *P*referred *R*eporting *I*tems for *S*ystematic Reviews and *M*eta-*A*nalyses: The PRISMA Statement. PLoS Med 6(6): e1000097. doi:10.1371/journal.pmed1000097. For more information, visit www.prisma-statement.org.

Using the search terms, we identified 1358 publications in the PubMed, Embase, and Cochrane databases and via the internet until November 2015. In the initial screening of these publications, 1334 articles were excluded because of the following reasons: 1) the determinants were not carotid arterial stiffness and the outcomes were not CV events, CV mortality or all-cause mortality, 2) review articles, letters, editorials, commentaries, guidelines or protocols, 3) *in-vitro* or *in-vivo* animal studies, or 4) case reports or other unrelated articles (Fig 1). Twenty-four relevant studies were identified, and their full-texts were reviewed for the second screening. Of the 24 studies, 13 studies were excluded because of the following reasons: 1) not a prospective study (n = 4), 2) abstracts only (n = 3), 3) component of a further study (n = 2), 4) published repeatedly (n = 1), 5) lacked data regarding carotid DC (n = 2), and 6) lacked data regarding the risk estimate for carotid DC (n = 1) (Fig 1). Finally, 11 studies with 20361 subjects were included in the meta-analysis. Of the 11 studies, 9 studies (18993 subjects) reported the risk estimates for CV events compared with 2 studies (2550 subjects) for CV mortality and 6 studies (3619 subjects) for all-cause mortality. The 11 studies were published from 1998 to 2014 and had a mean or median follow-up from 8.8 to 165 months and a sample size from 68 to 10470 subjects. The details of the 11 studies are shown in Table 1. In the quality assessment using the Newcastle-Ottawa Scale (NOS), the study quality scores varied from 5 to 9 points with a median of 7 points (Table 2).

**Table 1:** Details of the studies included in the meta-analysis.

Study	Population	Mean age (Years)	Gender ratio (% of male)	Duration of follow-up (Months)	Outcomes	Formula for calculate DC	Vascular bed	Imaging modality	Reliability	Mean stiffness parameters	Cut-off values	Stiffness parameters in models	Adjustments in models
Blacher et al., 1998 [5]	ESRD (n = 79)	58 ± 15	60%	25 ± 7	All-cause (n = 18) and CV mortality (n = 10).	DC = 2(ΔD/ D_d_) / ΔP	Stroke change of diameters of and pulse pressure of the right CCA	B-mode ultrasound in conjunction with a vessel wall track system (Neurodata)	Repeatability coefficient was ± 1 kPa^-1^·10^−3^ for DC	14.7 ± 7.2	≤9	Worst quartile *vs* other quartiles	DBP and total/HDL cholesterol ratio
Barenbrock et al., 2002 [6]	ESRD after renal transplantation (n = 68)	42 ± 2	57.4%	95 ± 2	CV events (n = 19) and CV mortality (n = 6)	DC = 2(ΔD/ D_d_) / ΔP	Stroke change of diameters of the left CCA and pulse pressure of the brachial artery	Ultrasound in conjunction with a vessel wall track system (University of Limburg); and a sphygmomanometer	Coefficient of Variation was 10.8% for DC.	15.5 ± 0.8	1 SD	Continuous	End-diastolic diameter, age, sex, smoking. systolic blood pressure, diastolic blood pressure, heart rate, haemodialysis period, serum creatinine, total serum cholesterol, and haemoglobin.
Stork et al., 2004 [11]	Elderly men (n = 367)	78 ± 4	100%	48	All-cause (n = 70) and cardiovascular mortality (n = 28).	DC = 2(ΔD/ D_d_) / ΔP	Stroke change of diameters of the right CCA and pulse pressure of the brachial artery of right upper arm	Ultrasound in conjunction with a vessel wall track system (Pie Medical); and be read automatically (Dinamap, Critikon)	Coefficient of Variation (Reproducibility) was 8.5% and 1.2% for D_d_ and ΔD respectively	9.68 ± 4.18	1 SD	Continuous	Age
Dijk ea al., 2005 [12]	Patients with manifest arterial disease (n = 2183)	59.7 ± 10.4	75%	Mean:33.6 (range: 1.2–78)	CV events (n = 192) and CV mortality (n = 107)	DC = 2(ΔD/ D_d_) / ΔP	Mean stroke change of diameters of the left and right CCA; and pulse pressure of the brachial artery	Ultrasound in conjunction with a wall track system	Intra-observer coefficient of Variation was 2.1% and 6.2% for D_d_ and ΔD respectively	14.1 ± 6.4	1 SD	Continuous	Age, gender, MAP, packyears smoked, and use of antihypertensive medication
Mattace-Raso et al., 2006 [13]	Population-based cohort (n = 2835, 2265 of them were include in analyses)	71.7 ± 6.7 for the total 2835 subjects	39.2% of men for the total 2835 subjects	64.8 ± 13.2	CV events (n = 124) and all-cause mortality (n = 265)	DC = 2(ΔD/ D_d_) / ΔP	Stroke change of diameters of the right CCA; and pulse pressure of the brachial artery of right upper arm	Ultrasound in conjunction with a vessel wall track system; and a random-zero sphygmomanometer	ICC = 0.8	10.6 ± 4.4	8.8 and 12.7 for the second and third tertiles in men and corresponding 7.8 and 11.3 in women	Tertile	Age, gender, arterial pressure, heart rate, body mass index, total cholesterol, HDL cholesterol, diabetes mellitus, smoking status, use of antihypertensive medication, carotid IMT, AAI, and pulse pressure.
Leone et al., 2008 [14]	Population-base cohort with subjects aged 65 years or above(n = 3337)	73.2 ± 4.7	39.4%	Median 43.4 (1–48)	CHD	DC = (ΔA/A_d_)/ΔP	Stroke change of diameters and pulse pressure of the CCA	Ultrasound in conjunction with an automated computerized system to determine the arterial diameter waveform	The ICC was 0.83 and 0.66 for Dd and carotid distension, respectively. The coefficient of variation (CV) was 5.7% for Dd and 14.2% for carotid distension.	28.39 ± 10.77	Upper tertile and 1 SD	Tertile and continuous	Age, sex, center, smoking status, BMI, MBP, heart rate, antihypertensive drugs, LDL cholesterol, log triglycerides, lipid lowering drugs, diabetes mellitus, cardiovascular diseases history, CCA-IMT, carotid plaques and educational level
Haluska et al., 2010 [7]	A cohort of patient with CV risk factors (n = 719)	55 ± 11	52%	57 ± 17	All-cause mortality	DC = 2(ΔD/ Dd) / ΔP	Stroke change of diameters of the CCA and blood pressure of brachial artery in the arm	B-mode ultrasound	Non-reported	22 ± 11	1 SD	Continuous	Framingham risk, BMI, central blood pressure, hemodynamic variables, and total arterial compliance
Karras et al., 2012 [8]	A subset of the NephroTest cohort of patient s with stages 2 to 5 CKD, but not yet dialysis (n = 439, 180 of them had carotid evaluation)	59.8 ± 14.5	74%	56.04 ± 10.2	Fatal and non-fatal CV events	((ΔA/A_d_)/ΔP)^-1/2^	Stroke change of diameters and pulse pressure of the right CCA	Ultrasound in conjunction with a vessel wall track system (Wall Track System, Esaote, The Netherlands)	Non-reported	18.3 ± 6.2	1 SD	Continuous	Non-adjusted
Yang et al., 2012 [9]	A subset of the Atherosclerosis Risk in Communities study (ARICn = 10470)	With CVE, 57.1 ± 5.7, n = 1547; without CVE, 55.0 ± 5.9, n = 8860.	With CVE, 60.8%, n = 1547; without CVE, 39.2%, n = 8860	Mean 165.6	Fatal and non-fatal CV events	DC = (ΔA/A_d_)/ΔP	Stroke change of diameters of the left CCA and blood pressure of brachial artery in the arm	Ultrasound in conjunction with a vessel wall track system	The correlation coefficient between measures was 0.67 for arterial distensibility.	17.4 ± 6.9	1 SD	Continuous	Age, gender, study site, race, height, weight, diabetes, total cholesterol, high-density lipoprotein cholesterol, smoking status, systolic blood pressure, antihypertensive medication use and carotid intima-media thickness.
van Sloten et al., 2014 [10]	A population based cohort (n = 579)	69.7 ± 6.3	49.4%	Median: 91.2 (2.4–106.8)	CV events and all-cause mortality	DC = (ΔA/A_d_)/ΔP	Stroke change of diameters of the right CCA and pulse pressure of the left brachial artery	M-mode ultrasound vessel wall movement detection software and an acquisition system (Wall Track System, Pie Medical)	Coefficients of variation were 7.0% for DC	10.83 ± 4.2	1 SD	Continuous	Age, gender, glucose metabolism status, arterial pressure, cardiovascular diseases, body mass index, triglycerides, total/HDL cholesterol ratio, estimated glomerular filtration rate, microalbuminuria, physical activity and smoking habits
Sung et al., 2014 [15]	A population of patient with possible structural or functional reasons for the development of heart failure (n = 114)	63.5 ± 17.5	73.7%	8.8 ± 3.5	Heart failure	DC = 2(ΔD/ D_d_) / ΔP	Stroke change of diameters of the right CCA and pulse pressure of the left brachial artery	B-mode ultrasound	Intra- and inter-observer ICCs: 0.986 and 0.943	21.0 ± 8.7	14.1	Quartile	Non-adjusted

ESRD, end-stage renal disease; CKD, chronic kidney disease; CV, cardiovascular; DC, distensibility coefficient, kPa^-1^·10^−3^; CC, compliance coefficient, mm^2^/kPa; CCA, common carotid artery; ΔD, diameters of arterial distension; D_d_, diameter in diastole; ΔA, cross-sectional area of arterial distension; A_d_; cross-sectional area in diastole; ΔP, pulse pressure.

**Table 2:** Assessment of study quality using the Newcastle-Ottawa Quality Assessment Scale.

Studies	Selection				Comparability		Outcome			Total Scores
	1	2	3	4	5	6	7	8	9	
Blacher et al., 1998 [5]	**✵**	**✵**	**✵**	**✵**	-	**✵**	**✵**	-	**✵**	7
Barenbrock et al., 2002 [6]	**✵**	**✵**	**✵**	**✵**	**✵**	-	**✵**	**✵**	**✵**	8
Stork et al., 2004 [11]	**✵**	**✵**	**✵**	**✵**	**✵**	-	**✵**	-	**✵**	7
Dijk et al., 2005 [12]	**✵**	**✵**	**✵**	-	**✵**	**✵**	**✵**	-	**✵**	7
Mattace-Raso et al., 2006 [13]	**✵**	**✵**	**✵**	**✵**	**✵**	**✵**	**✵**	**✵**	**✵**	9
Leone et al., 2008 [14]	**✵**	**✵**	**✵**	-	**✵**	**✵**	**✵**	-	**✵**	7
Haluska et al., 2010 [7]	**✵**	**✵**	**✵**	**✵**	-	**✵**	**✵**	-	**✵**	7
Karras et al., 2012 [8]	**✵**	**✵**	**✵**	-	-	-	**✵**	-	**✵**	5
Yang et al., 2012 [9]	**✵**	**✵**	**✵**	**✵**	**✵**	**✵**	**✵**	**✵**	**✵**	9
van Sloten et al., 2014 [10]	**✵**	**✵**	**✵**	-	**✵**	**✵**	**✵**	**✵**	**✵**	8
Sung et al., 2014 [15]	**✵**	**✵**	**✵**	**✵**	-	-	**✵**	-	**✵**	6

1. Representativeness of the exposed cohort.

2. Selection of the non-exposed cohort.

3. Ascertainment of exposure.

4. Demonstration that the outcome of interest was not present at study initiation.

5 and 6. Comparability of cohorts on the basis of the design or analysis (Studies that controlled for age were assigned one score, and studies that controlled for other critical covariates were assigned another score).

7. Assessment of outcome.

8. Sufficient follow-up for outcomes to occur (Studies with a follow-up duration more than 5 years were assigned one score).

9. Adequacy of cohorts follow up (Studies with complete follow-up or subjects lost to follow up unlikely to introduce bias were assigned one score).

### Meta-analysis for CV events

**Fig 2:**
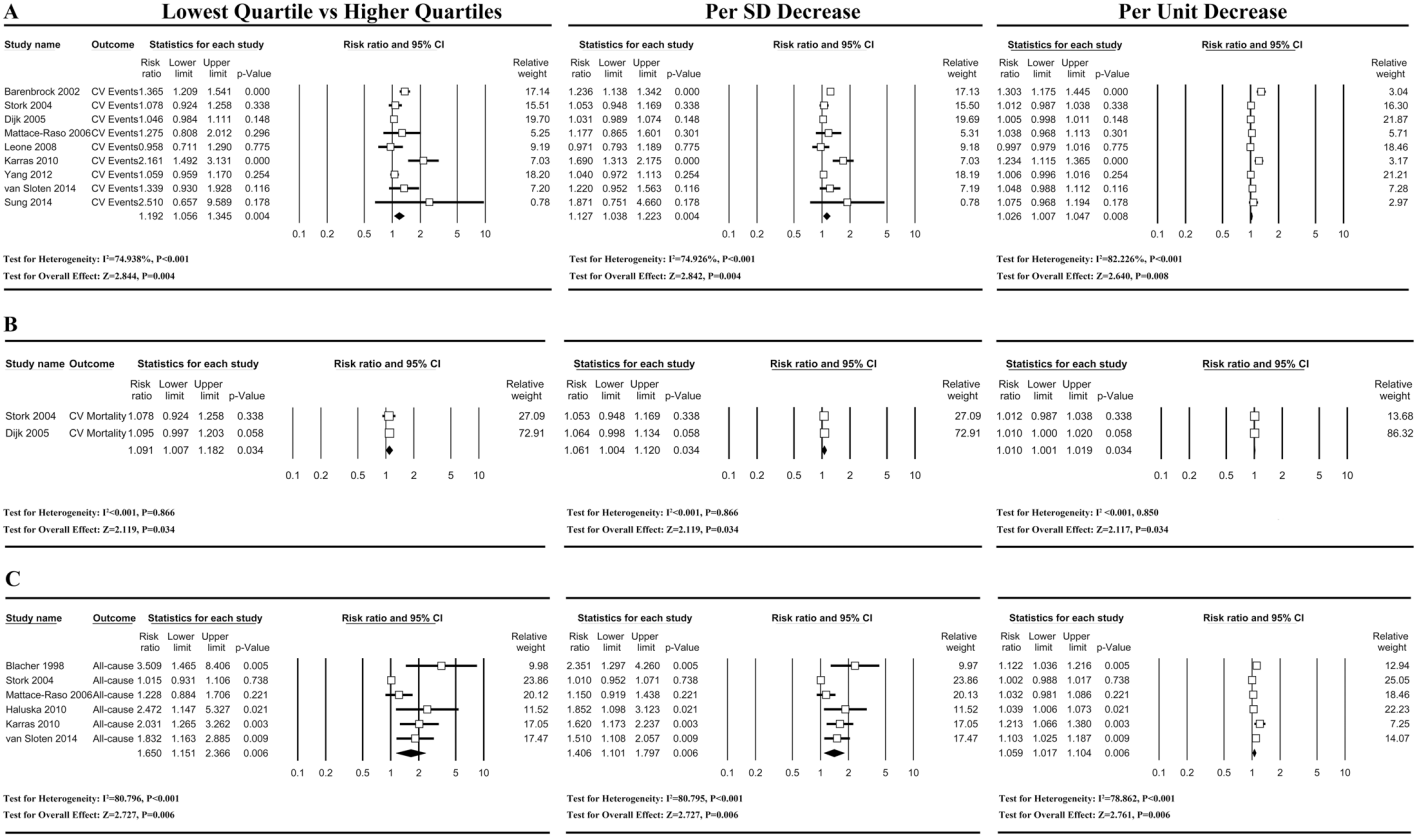
RR and 95%CI for low carotid DC and clinical events. Risk ratios (RRs) and 95% confidence intervals (CIs) of cardiovascular (CV) events (**A**), CV mortality (**B**) and all-cause mortality (**C**) for a low carotid distensibility coefficient (DC). Open boxes mean the RRs, and lines indicate the 95% CI for individual studies; solid diamonds represent the pooled RRs, and their widths show the pooled 95%CI.

For CV events, 9 studies were included in the meta-analysis. The individuals with a lower DC had a significantly increased risk of CV events when compared with their counterparts with a greater DC (P<0.05). The pooled RR of CV events was 1.19 (1.06–1.35, 95%CI) for the lowest quartile compared with the higher quartiles of DC. For the 1 SD decrease and 1 unit decrease of DC, the pooled RRs were 1.13 (1.04–1.22, 95%CI) and 1.03 (1.01–1.05, 95%CI), respectively (Fig 2A). Furthermore, in the studies with a high-risk population (n = 6, ESRD patients [6, 8], elderly individuals [11, 14], patients with manifest arterial disease [12] or patients with potential heart failure [15]), the corresponding pooled RRs of CV events were slightly increased to 1.23 (1.03–1.87, 95%CI), 1.15 (1.02–1.30, 95%CI), and 1.05 (1.01–1.09, 95%CI), respectively. In the sub-group analysis for stroke [9, 12, 13] and CHD [9, 12–14], the pooled RRs were 1.08 (0.96–1.22, 95%CI) and 1.02 (0.98–1.07, 95%CI) for a 1 SD decrease in DC, respectively. When including the unpublished and up-to-date data (unpublished data of 579 subjects with an event rate of 0.9% and 1.3% and a median follow-up duration of 7.7 and 7.7 years, respectively, for stroke and CHD in the study of van Stolen et al. [10], as well as unpublished up-to-date data of 4713 subjects with an event rate of 0.8% and 1.2% and a median follow-up duration of 12 and 9.9 years, respectively, for stroke and CHD in the study of Mattace-Raso et al. [13]), the pooled RRs of stroke and CHD were 1.15 (1.01–1.30) and 1.03 (0.99–1.07), respectively (S1 Fig). Thus, carotid DC significantly predicted stroke but not CHD.

**Fig 3:**
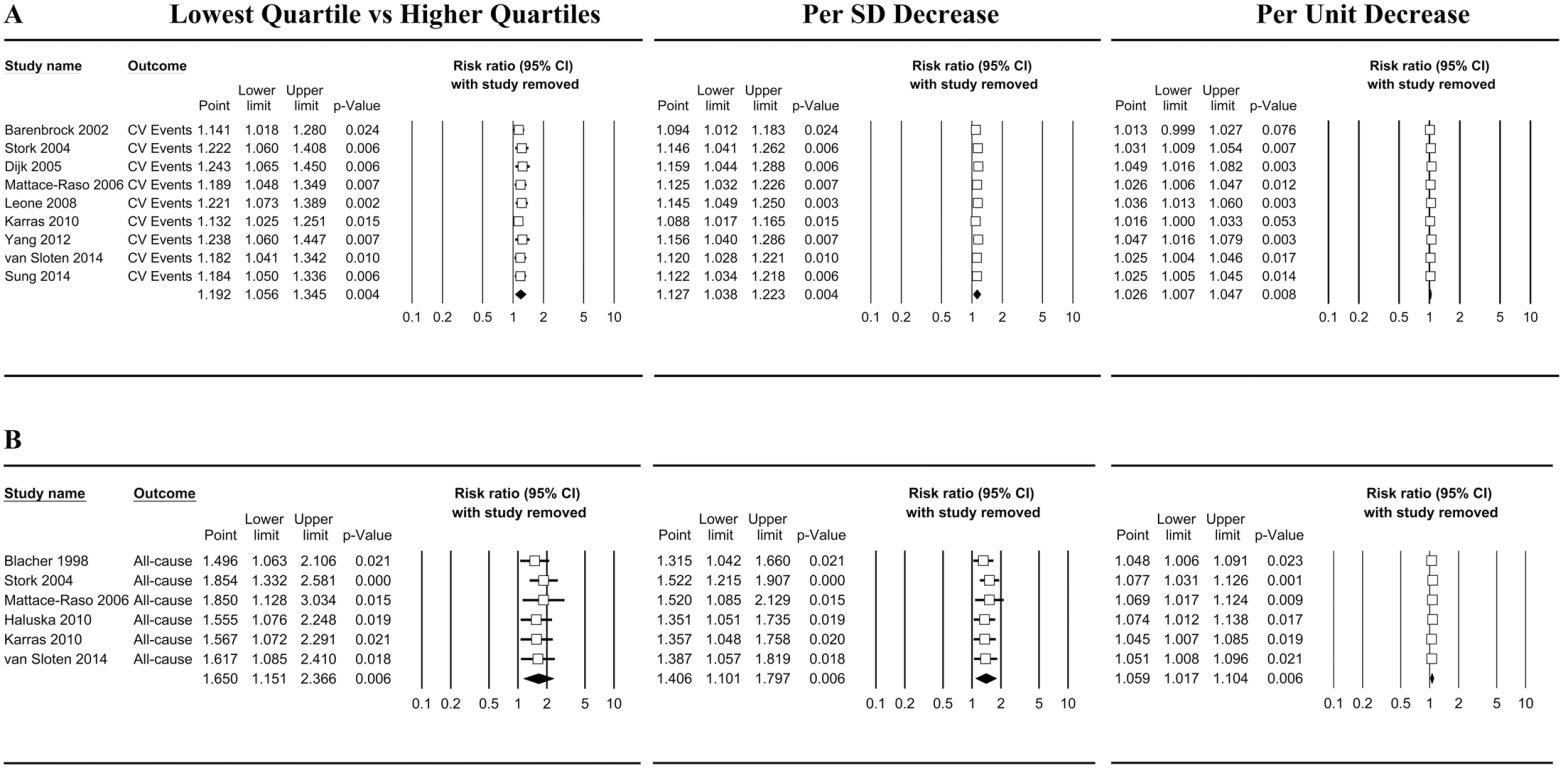
Sensitivity analysis for CV events and all-cause mortality. Sensitivity analysis for cardiovascular (CV) events (**A**) and all-cause mortality (**B**). Open boxes mean the summary RRs, and lines indicate the summary 95% CI when that row’s study is removed from the meta-analysis; solid diamonds represent the pooled RRs, and their widths show the pooled 95%CI when the meta-analysis includes all studies.

The sensitivity analysis indicated that, for CV events, the exclusion of a single study did not alter the final result, with pooled RRs that ranged from 1.14 (1.02–1.28, 95%CI) to 1.24 (1.07–1.48, 95%CI) for the lowest quartile, 1.09 (1.02–1.17, 95%CI) to 1.16 (1.04–1.29, 95%CI) for a 1 SD decrease, and 1.01 (1.00–1.03, 95%CI) to 1.05 (1.02–1.08, 95%CI) for a 1 unit decrease in DC (Fig 3A). In addition, when the studies with a follow-up duration less than 5 years [11, 12, 14, 15], an NOS score less than 7 points [8, 15], or a RR derived from OR [6] were excluded, carotid DC continued to significantly predict future CV events (P<0.05, S3 Table). Thus, the duration of follow-up, NOS score and OR-derived RR did not change the significance of carotid DC in the prediction of CV events.

**Fig 4:**
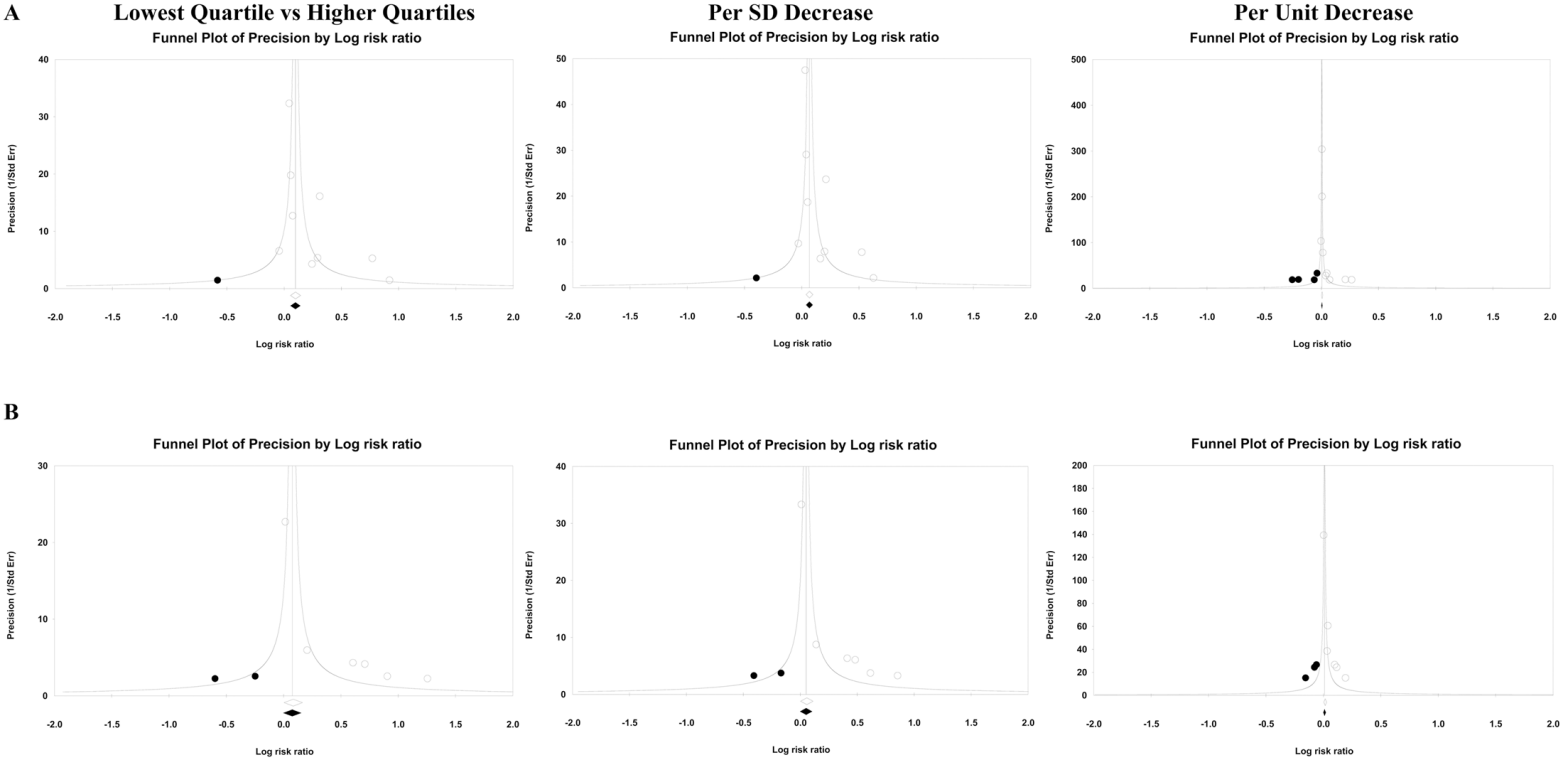
Publication bias for CV events and all-cause mortality. Funnel plots of the precision for cardiovascular (CV) events (**A**) and all-cause mortality (**B**). Open circles indicate individual studies in the correlation of carotid distensibility coefficient with events, and open diamonds mean the risk ratios and 95%CI for the meta-analysis. Solid circles indicate the imputed studies, and solid diamonds mean the risk ratios and 95%CI after adjustments for publication bias.

The publication bias analysis demonstrated that the funnel plot was symmetrically distributed at both the left and right sides of the axis (Fig 4A), which indicated that the quantity of the studies with negative or positive risk estimates was similar. Furthermore, the imputed RRs of CV events were 1.18 (1.05–1.34, 95%CI) and 1.12 (1.03–1.21, 95%CI) for the lowest quartile and a 1 SD decrease in DC, respectively. The imputed RRs were lower than the original risk estimates; however, they were still significant. For the 1 unit decrease in DC, the imputed RR was 1.01 (0.99–1.02, 95%CI), which was insignificant. In addition, the fail-safe N test indicated that the number of missing studies that would produce insignificant result was 61, which indicates that 6.8 (61/9) unpublished or undiscovered studies for every one study in our meta-analysis may change the significant results. Thus, the publication bias findings suggested that unpublished or undiscovered studies, if any, were insufficient to affect our findings regarding the lowest quartile and the per SD decrease in DC in CV event prediction.

### Meta-analysis for CV mortality

Only 2 studies assessed the risk of CV mortality for a lower DC. The pooled RRs of CV mortality for the lowest quartile, a 1 SD decrease, and a 1 unit decrease in DC were 1.09 (1.01–1.18, 95%CI), 1.06 (1.00–1.12, 95% CI), and 1.01 (1.00–1.02, 95%CI), respectively (Fig 2B). Two studies had high-risk populations (elderly individuals [11] and patients with manifest arterial disease [12]). However, the publication bias and the sensitivity cannot be assessed in two studies, although a meta-analysis may include only two studies [27]. Thus, it is possible that a negative risk estimate, if any, in unpublished or undiscovered data or studies may influence the significant pooled RRs.

### Meta-analysis for all-cause mortality

Six studies reported the risk estimates of all-cause mortality and were included in the meta-analysis. The risk of all-cause mortality for the lowest quartile, a 1 SD decrease, and a 1 unit decrease in DC were significant with pooled RRs of 1.65 (1.15–2.37, 95%CI), 1.41 (1.10–1.80, 95%CI), and 1.06 (1.02–1.10, 95%CI), respectively (Fig 2C). In high-risk populations (n = 4, ESRD patients [6, 8], elderly individuals [12], and patients with cardiovascular risk factors [7]), the risk estimates were correspondingly increased to 1.89 (1.02–3.51, 95%CI), 1.54 (1.01–2.35, 95%CI), and 1.06 (1.01–1.12, 95%CI), respectively.

The sensitivity analysis demonstrated that the pooled RRs were not altered when a single study was removed, with pooled RRs that ranged from 1.50 (1.06–2.11, 95%CI) to 1.85 (1.33–2.58, 95%CI) for the lowest quartile, 1.31 (1.04–1.66, 95%CI) to 1.52 (1.22–1.90, 95%CI) for a 1 SD decrease, and 1.05 (1.01–1.09, 95%CI) to 1.08 (1.03–1.13, 95%CI) for a 1 unit decrease in DC (Fig 3B). In addition, the exclusion of the studies with a follow-up duration less than 5 years [5, 7, 11], an NOS score less than 7 points[8], or a RR derived from OR [5] did not alter the significant findings (P<0.05) (S3 Table).

Regarding the publication bias of the six studies, the funnel plot was asymmetrically distributed at the bottom, which indicated that small studies with small or negative risk estimates were missed in the present meta-analysis (Fig 4B). Using the trim-and-fill method to impute the missing studies, the adjusted risk estimates remained significant for the lowest quartile (1.40, 1.02–1.93, 95%CI) and a 1 SD decrease (1.26, 1.01–1.56, 95%CI), but not for a 1 unit decrease in DC (1.02, 0.98–1.06, 95%CI). In addition, the fail-safe N test indicated that the number of missing studies that would produce insignificant results was 33, which suggests that 5.5 (33/6) unpublished or undiscovered studies for every one study included in the present meta-analysis may change the significant results. Thus, the publication bias was insufficient to affect the predictive value of the lowest quartile and the per SD decrease in DC for all-cause mortality.

## Discussion

The present study investigated the predictive value of carotid DC for future CV diseases and all-cause mortality. In the meta-analysis, 20361 individuals from 11 longitudinal studies were pooled. The individuals with the lowest quartile of DC had 1.19 times the risk of CV events, 1.09 times the risk of CV mortality and 1.65 times the risk of all-cause mortality compared with the individuals with the 3 higher quartiles of DC. A decrease in DC of 1 SD predicted a 13%, 6%, and 41% higher incidence of CV events, CV mortality and all-cause mortality, respectively, whereas a decrease in DC of 1 unit predicted a 3%, 1% and 6% higher incidence of the corresponding clinical events. Moreover, the predictive value of carotid DC for CV events and all-cause mortality was stronger in the high-risk population.

These findings were consistent with the results from another meta-analysis, which was published during the review process of our study [28]. Based on the published and unpublished data, as well as the recalculated risk estimates, van Sloten et al. demonstrated that a 1 SD greater carotid stiffness significantly predicted stroke (1.18, 1.05–1.33, 95%CI), CV events (1.16, 1.07–1.26, 95%CI), CV mortality (1.30, 1.15–1.46, 95%CI), and all-cause mortality (1.22, 1.12–1.34, 95%CI), but not CHD (1.03, 0.98–1.10, 95%CI). Interestingly, they used YEM instead of DC in their meta-analysis when including the studies of Dijk et al., Leone et al., and Stork et al. (with the reason that the risk estimates for DC were not available). However, the risk estimates for DC were indeed reported in the three publications [11, 12, 14]. Various stiffness parameters may have different values for the prediction [11, 15], and may be in caution for combination. In addition, they did not investigate the risk estimates for other categories of DC or in different populations. In a separate analysis for DC (which did not include the studies of Dijk et al., Leone et al., and Stork et al.), van Sloten et al. reported that the pooled HRs of CV events, CV mortality and all-cause mortality were 1.14 (1.04–1.26, 95%CI), 1.47 (1.05–2.06, 95%CI) and 1.30 (1.11–1.53, 95%CI), respectively, for a 1 SD lower DC. We recalculated the risk estimates in their meta-analysis for various categories of DC and in different populations. The HRs of CV events, CV mortality and all-cause mortality were 1.22 (1.06–1.40, 95%CI), 1.77 (1.07–2.90, 95%CI) and 1.47 (1.16–1.87, 95%CI), respectively, for the lowest quartile and 1.03 (1.00–1.06, 95%CI), 1.05 (1.01–1.10, 95%CI) and 1.04 (1.02–1.06, 95%CI), respectively, for a 1 unit decrease. In the high-risk population, the corresponding risk estimates were increased to 1.42 (1.02–1.99, 95%CI), 2.31 (1.25–4.27, 95%CI) and 1.80 (1.13–2.85, 95%CI) for the lowest quartile, 1.27 (1.01–1.61, 95%CI), 1.77 (1.16–2.69, 95%CI) and 1.49 (1.09–2.04, 95%CI) for a 1 SD decrease, and 1.06 (1.01–1.10, 95%CI), 1.07 (1.02–1.13, 95%CI) and 1.05 (1.01–1.09, 95%CI) for a 1 unit decrease in DC. Thus, the two independent meta-analyses both indicated that carotid DC significantly predicts CV diseases and all-cause mortality, and the prediction is stronger when the lowest quartile of DC and a high-risk population are considered.

For the 20361 subjects from the 11 studies in the present meta-analysis, the aggregate DC was 17.9 ± 7.2 kPa^-1^·10^−3^. As a result, the lowest quartile of DC should be 13.0 kPa^-1^·10^−3^, which is equal to a local PWV of 8.8 m/s (PWV = DC^-1/2^, when assuming the blood density as 1 g/cm^3^ [3]). Nevertheless, local carotid PWV cannot be directly used as aortic PWV. Paini et al. demonstrated that the stiffening of the carotid artery was lower compared with the aortic artery with age and CV risk factors [29]. In four studies that reported both carotid DC and aortic PWV [8, 10, 13, 30], local carotid PWV (derived from DC) was 3.2 m/s lower than aortic PWV (3088 subjects). Therefore, 13 kPa^-1^·10^−3^ in carotid DC (equal to 8.8 m/s in carotid PWV) may represent 12 m/s in aortic PWV to some extent. In current clinical practice, 12 m/s is recommended as the cut-off value of aortic PWV for the stratification of high-risk patients [31, 32]. Thus, 13.0 kPa^-1^·10^−3^ may be used as the cut-off value of carotid DC for the stratification of patients who have an increased risk of CV diseases and may require follow-up more closely. In addition, carotid DC is capable of predicting stroke and all-cause mortality independent of CV risk factors and aortic PWV [28]. Moreover, carotid DC additionally improves the risk prediction beyond CV risk factors and aortic PWV [28]. Aortic PWV is currently used to stratify high-risk patients in the management of arterial hypertension, and no practice guideline has documented the use of carotid DC in clinics[32, 33]. However, the assessment of aortic PWV is not always available because of limited devices and specific patients [2, 3]. In contrast, the measurement of carotid DC is more available because it can be assessed using two widely available imaging modalities, ultrasound and MRI, and, for most patients, via the measurement of the pulsatile motions of the carotid artery wall. Thus, carotid DC may be used for the prediction of CV diseases independently and additionally regardless of whether the measurement of aortic PWV is available.

Nevertheless, the predictive value of carotid DC was not as strong as aortic PWV [17]. Vlachopoulos et al. reviewed the predictive value of aortic PWV for future CV events and all-cause mortality in 17 longitudinal studies; the results indicated that the pooled RRs of CV events, CV mortality and all-cause mortality were 2.26 (1.89–2.7, 95%CI), 2.02 (1.68–2.42, 95%CI) and 1.9 (1.61–2.24, 95%CI), respectively, in the comparison of higher and lower aortic PWVs [17]. In addition, a 1 SD increase in aortic PWV indicated an increase of 47%, 47% and 42% for CV events, CV mortality and all-cause mortality, respectively. The weaker predictive value of carotid DC may occur, in part, because the peripheral BP and simplified formula were used for the calculation of carotid DC in most studies. DC should be calculated using the following formula: DC = (ΔA/Ad)/ΔP = (2ΔD×Dd+ΔD^2^)/ (ΔP×Dd^2^), in which ΔA is the cross-sectional area of the arterial distension, Ad means the cross-sectional area in diastole, ΔP shows the carotid pulse pressure (PP), ΔD indicates the diameter of the arterial distension, and Dd represents the diameter in diastole. Eight of the 11 studies used peripheral BP to represent local carotid BP for calculating DC. There are changes in the amplitude and timing of wave reflection along the arterial tree; thus, SBP measured at the brachial artery is commonly increased compared with the carotid artery [2]. The use of peripheral BP to calculate carotid DC may overestimate carotid PP, which consequently leads to an underestimation of the association between carotid DC and CV diseases. In addition, a simplified formula, DC = 2(ΔD/Dd)/ ΔP, was used in most studies (n = 7), which may cause an underestimation of the difference between the stiff and elastic carotid arteries (especially when ΔD is large compared with Dd) [3] and may consequently underestimate the predictive value of DC for CV diseases.

In conclusion, carotid DC is a significant predictor for future CV diseases and all-cause mortality, although the predictive value is not as strong as aortic PWV. In clinics, the lowest quartile of carotid DC may be used as a cut-off value to stratify patients who have an increased risk of future CV events, especially in populations of elderly individuals and patients with CV risk factors, so as to conduct an early diagnosis and prompt treatment. Nevertheless, future studies are needed to identify the cut-off value in different countries and races, investigate the effectiveness of the cut-off value for the prediction, and determine a strategy for follow-up to balance the costs and benefits epidemiologically.

## Limitations

A small number of studies were available and included in the present meta-analysis (especially regarding the CV mortality, in which only two studies were included in the analysis), which limited the publication consistency and our findings. In addition, the definitions of CV events in the included studies were different, which may introduce bias factors in the present study. Furthermore, local PP rather than peripheral PP may be used for the precise measurement of carotid DC; however, these variables were not fully investigated in the present study. Future studies remain to be conducted to address these problems.

## Supporting Information

S1 Fig Subgroup analysis for stroke and coronary heart diseases. Pooled RRs of stroke (A) and coronary heart disease (B) with published data (left) or with unpublished up-to-date data (right). Open boxes mean the RRs, and lines indicate the 95% CI for individual studies; solid diamonds represent the pooled RRs, and their width shows the pooled 95%CI.
(TIF)

S1 Table Definitions of outcomes in the studies included in the present meta-analysis. (DOCX)

S2 Table Data extraction and conversion. (DOCX)

S3 Table Sensitivity analyses by removing unfavorable studies. (DOCX)

S4 Table PRISMA 2009 Checklist. (DOC)
