# Supplementary figures and images for: Predictive Value of Carotid Distensibility Coefficient for Cardiovascular Diseases and All-Cause Mortality: A Meta-Analysis
Source: PLoS One. 2016 Apr 5;11(4):e0152799. doi: 10.1371/journal.pone.0152799 (PMC4821582; doi:10.1371/journal.pone.0152799)

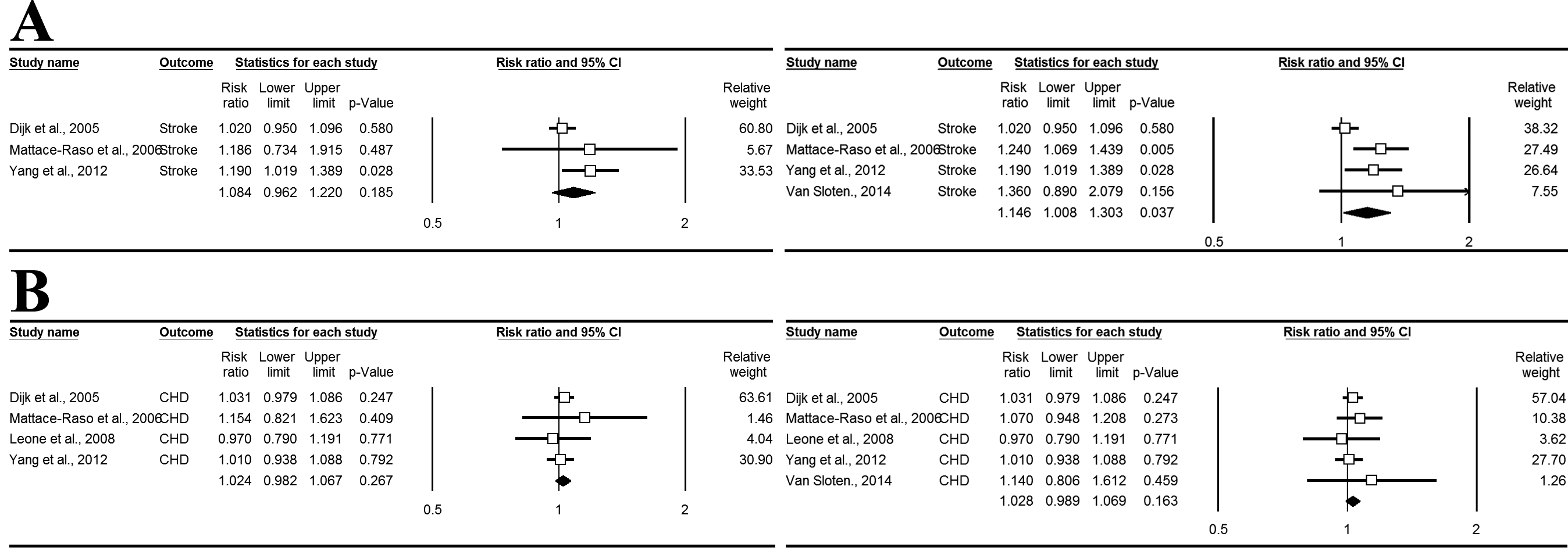

Supplement: S1 Fig — Pooled RRs of stroke (A) and coronary heart disease (B) with published data (left) or with unpublished up-to-date data (right). Open boxes mean the RRs, and lines indicate the 95% CI for individual studies; solid diamonds represent the pooled RRs, and their width shows the pooled 95%CI. (TIF) [file pone.0152799.s001.tif]
