# Supplementary material for: Predictive Value of Carotid Distensibility Coefficient for Cardiovascular Diseases and All-Cause Mortality: A Meta-Analysis
Source: PLoS One. 2016 Apr 5;11(4):e0152799. doi: 10.1371/journal.pone.0152799 (PMC4821582; doi:10.1371/journal.pone.0152799)
Supplement: S1 Table — (DOCX) [file pone.0152799.s002.docx]

**S1 Table. Definitions of outcomes in the studies included in the present meta-analysis**

| Studies | Definition of outcome | Outcomes |
| --- | --- | --- |
| Blacher et al., 1998 [5] | Stroke, CHD, HF and sudden death | All-cause mortality |
| Barenbrock et al., 2002 [6] | Stroke, CHD, PAD and HF | CV events |
| Stork et al., 2004 [11] | Stroke, CHD and HF | CV events, and CV and all-cause mortality |
| Dijk ea al., 2005 [12] | Stroke (ischemic), CHD (MI), PAD and sudden death | CV events and CV mortality |
| Mattace-Raso et al., 2006 [13] | Stroke (ischemic or hemorrhagic), CHD (MI, CABG, or PTCA), and sudden death | CV events and all-cause mortality |
| Leone et al., 2008 [14] | CHD (MI, CABG, PTCA, or AP) and sudden death | CV events |
| Haluska et al., 2010 [7] | HF | CV events |
| Karras et al., 2012 [8] | Stroke, CHD, HF and PAD | CV events and all-cause mortality |
| Yang et al., 2012 [9] | Stroke (ischemic), CHD (MI, CABG, or PTCA), and sudden death | CV events |
| van Sloten et al., 2014 [10] | Stroke (ischemic), CHD (MI, CABG, PTCA, or AP), HF, PAD, and sudden death | CV events and all-cause mortality |
| Sung et al., 2014 [15] | Stroke, CHD (MI), HF and AP | All-cause mortality |

CHD, coronary heart disease; MI, myocardial infarction; CABG, coronary artery bypass grafting; PTCA, percutaneous transluminal coronary angioplasty; AP, angina pectoris; HF, heart failure; PAD, peripheral arterial disease.
