# Supplementary material for: Predictive Value of Carotid Distensibility Coefficient for Cardiovascular Diseases and All-Cause Mortality: A Meta-Analysis
Source: PLoS One. 2016 Apr 5;11(4):e0152799. doi: 10.1371/journal.pone.0152799 (PMC4821582; doi:10.1371/journal.pone.0152799)
Supplement: S2 Table — (DOCX) [file pone.0152799.s003.docx]

**S2 Table. Data extraction and conversion**

| Data Source | Original Data | Conversion Methods | RR for lowest versus higher quartiles | RR for per SD decrease | RR for per unit decrease |
| --- | --- | --- | --- | --- | --- |
| Blacher et al., 1998: Table 4 | OR of all-cause mortality: 6.4 (1.8-23.3, lowest vs higher quartiles) and P0=9/59 | RR=OR/((1-P0)+(OR×P0)) | RR^3^=3.51(1.47-8.41) | RR^3^=2.35(1.30-4.26) | RR^3^=1.12(1.04-1.22) |
| Barenbrock et al., 2002: Table 3 and Fig 1 | For 1 SD increase in DC, correlation coefficient β for CV events: -0.239 and t value: 5.014. P0= 4/39* | OR=Exp (-β)  SE=β/t  95%CI=Exp(β ± 1.96SE)  RR=OR/((1-P0)+(OR×P0)) | RR^1^=1.36 (1.21-1.54) | RR^1^=1.24(1.14-1.34) | RR^1^=1.30(1.17-1.44) |
| Stork et al., 2004: Table 2 | HRs of CV and all-cause mortality: 0.95 (0.86-1.06, 1 SD increase) and 0.99 (0.93-1.05, 1 SD increase), respectively | RR _increase_=HR  RR _decrease_=1/ RR _increase_ | RR^1^=1.08(0.92-1.26)  RR^2^=1.08(0.92-1.26)  RR^3^=1.01(0.93-1.11) | RR^1^=1.05(0.95-1.17)  RR^2^=1.05(0.95-1.17)  RR^3^=1.01(0.95-1.07) | RR^1^=1.01(0.99-1.04)  RR^2^=1.01(0.99-1.04)  RR^3^=1.00 (0.99-1.02) |
| Dijk ea al., 2005: Table 3 | HRs of CV events and CV mortality: 0.97 (0.93-1.01, 1 SD increase) and 0.94 (0.88-1.00, 1 SD increase), respectively | RR _increase_=HR  RR _decrease_=1/ RR _increase_ | RR^1^=1.05(0.98-1.11)  RR^2^=1.10(0.997-1.20) | RR^1^=1.03(0.99-1.07)  RR^2^=1.06(0.998-1.13) | RR^1^=1.00 (0.998-1.01)  RR^2^=1.01(0.9997-1.02) |
| Mattace-Raso et al., 2006: Table 3 | HRs of CV events and all-cause mortality: 1.37 (0.75-2.47, lowest vs higher tertiles) and 1.31 (0.85-2.03, lowest vs higher tertiles), respectively | RR=HR | RR^1^=1.28(0.80-1.99)  RR^3^=1.23(0.88-1.71) | RR^1^=1.18(0.86-1.60)  RR^3^=1.15(0.92-1.44) | RR^1^=1.04(0.97-1.11)  RR^3^=1.03(0.98-1.09) |
| Leone et al., 2008: Table 3 | HRs of CHD: 1.03 (0.84-1.26, 1 SD increase) | RR _increase_=HR  RR _decrease_=1/ RR _increase_ | RR^1^=0.96(0.71-1.29) | RR^1^=0.97(0.79-1.19) | RR^1^=1.00(0.98-1.02) |
| Haluska et al., 2010: Results | HRs of all-cause mortality: 0.54 (0.32–0.91, 1 SD increase) | RR _increase_=HR  RR _decrease_=1/ RR _increase_ | RR^3^=2.47(1.15-5.33) | RR^3^=1.85(1.10-3.12) | RR^3^=1.04(1.01-1.07) |
| Yang et al., 2012: Table S2 | HR of CV events: 1.04 (0.97-1.11, 1 SD decrease) | RR=HR | RR^1^=1.06(0.96-1.17) | RR^1^=1.04(0.97-1.11) | RR^1^=1.01(0.996-1.02) |
| van Sloten et al., 2014: Table 2 | HRs of CV events and all-cause mortality: 1.22 (0.95-1.56, 1 SD decrease) and 1.51 (1.11-2.06, 1 SD decrease), respectively | RR=HR | RR^1^=1.34(0.93-1.92)  RR^3^=1.83(1.17-2.89) | RR^1^=1.22(0.95-1.56)  RR^3^=1.51(1.11-2.06) | RR^1^=1.05(0.99-1.11)  RR^3^=1.10(1.03-1.19) |
| Sung et al. 2014: Table 4 | HR of heart failure: 2.51 (0.67-9.34, lowest vs higher quartiles) | RR=HR | RR^1^=2.51(0.67-9.34) | RR^1^=1.87(0.74-4.58) | RR^1^=1.07(0.97-1.19) |

P0, proportion of events in the reference group

*P0: in Fig 2B of Barenbrock’s study, the reference group had 4 events and a survival rate of 89%, whereas the exposed group had 15 events and a survival rate of 48%. When the total sample size was 68, the P0 was 4 events divided by 39.

RR increase, RR of outcome for the increase in carotid DC;

RR decrease, RR of outcome for the decrease in carotid DC;

RR1, RR of cardiovascular events for the lower carotid DC;

RR2, RR of cardiovascular mortality for the lower carotid DC;

RR3, RR of all-cause mortality for the lower carotid DC.

RR, risk ratio; OR, odds ratio; HR, hazard ratio; SD, standard deviation; DC, distensibility coefficient; CV, cardiovascular.

x=z(σ)+µ was used to calculate the percentile of DC, in which z is the z score of a specific certain percentile when a normal distribution of a value is assumed (http://www.measuringu.com/zcalcp.php), µ is the mean and σ is the SD,
