# Supplementary material for: Predictive Value of Carotid Distensibility Coefficient for Cardiovascular Diseases and All-Cause Mortality: A Meta-Analysis
Source: PLoS One. 2016 Apr 5;11(4):e0152799. doi: 10.1371/journal.pone.0152799 (PMC4821582; doi:10.1371/journal.pone.0152799)
Supplement: S3 Table — (DOCX) [file pone.0152799.s004.docx]

**S3 Table. Sensitivity analyses by removing unfavorable studies.**

|  | Outcomes | Pooled RRs including all eligible studies | Pooled RRs following the removal of studies with a follow-up duration less than 5 years | Pooled RRs following the removal of studies with an NOS score less than 7 points | Pooled RRs following the removal of studies with an OR-derived RR |
| --- | --- | --- | --- | --- | --- |
| Lowest quartile | CV events | 1.19 (1.06-1.35) | 1.35 (1.09-1.67) | 1.13 (1.02-1/24) | 1.14 (1.02-1.28) |
|  | All-cause mortality | 1.65 (1.15-2.37) | 1.60 (1.16-2.21) | 1.57 (1.07-2.29) | 1.50 (1.06-2.11) |
| Per SD decrease | CV events | 1.13 (1.04-1.22) | 1.23 (1.06-1.42) | 1.09 (1.01-1.16) | 1.09 (1.01-1.18) |
|  | All-cause mortality | 1.40 (1.10-1.80) | 1.38 (1.10-1.72) | 1.36 (1.05-1.76) | 1.32 (1.04-1.66) |
| Per unit decrease | CV events | 1.03 (1.01-1.05) | 1.11 (1.02-1.20) | 1.02 (1.00-1.03) | 1.01 (1.00-1.03) |
|  | All-cause mortality | 1.06 (1.02-1.10) | 1.10 (1.01-1.19) | 1.05 (1.01-1.09) | 1.01 (1.00-1.03) |

CV, cardiovascular; SD, standard deviation; RR, risk ratio; OR, odds ratio; NOS, Newcastle-Ottawa Scale.
